# Mature Cystic Teratoma of the Pancreas: A Rare Cystic Neoplasm

**DOI:** 10.1515/med-2019-0102

**Published:** 2019-11-20

**Authors:** HeeJoon Kim, YangSeok Koh

**Affiliations:** 1 Department of General Surgery, Chonnam National University Hwasun Hospital, 58128 Hwasun-gun, South Korea

**Keywords:** Mature cystic teratoma, Pancreas, Laparoscopic distal pancreatectomy

## Abstract

Mature cystic teratoma of the pancreas is an extremely rare benign neoplasm. Only 51 cases have been reported in the literature. Its cystic nature often appears to have malignant potential in preoperative image studies. Moreover, no characteristic features could be shown on image studies, such as abdominal CT scan or pancreas MRI. The accurate diagnosis is generally obtained after surgical resection. We present a rare case of a 53-year-old male with mature cystic teratoma of the pancreas, which was confirmed on pathology after laparoscopic distal pancreatectomy.

## 1 Introduction

Primary mature cystic teratoma is a rare benign tumor, which is included in well-differentiated parenchymal tissues [1]. It is typically found in the ovary and testes, but it is very rare in the pancreas [2] and only 51 cases have been reported in the literature [3, 4, 5, 6]. Therefore, preoperative diagnosis is challenging due to no characteristic image findings [7]. Here, we report the 52nd case of a mature cystic teratoma of the pancreas confirmed on pathology after laparoscopic pancreatectomy in a 53-year-old male.

## 2 Case Report

**Figure 1:**
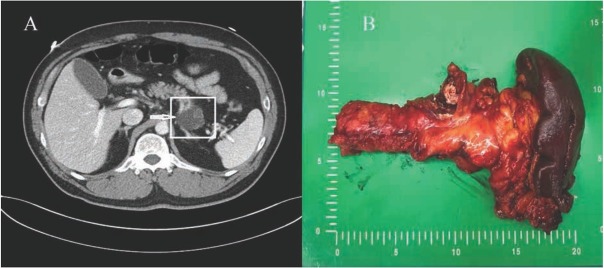
A. Abdominal CT scan showing a 4.4 x 4 cm-sized well-circumscribed multi-separated cystic mass (white arrow) in the pancreas tail with irregular enhancement in the inner septa.B. Specimen: 4 x 4 cm-sized cystic mass in the pancreas tail.

A 53-year-old man with a history of hypertension presented with an incidentally-detected cystic mass in the pancreas tail in the absence of abdominal complaints. Laboratory findings showed normal values including CEA and CA 19-9 levels. Pancreas MRI showed a 4.4 x 4.0 cm-sized well-circumscribed cystic mass arising from the pancreas tail with internal septa and delayed enhancement in peripheral portion (Fig. 1), suggesting a mucinous cystic neoplasm.

**Figure 2:**
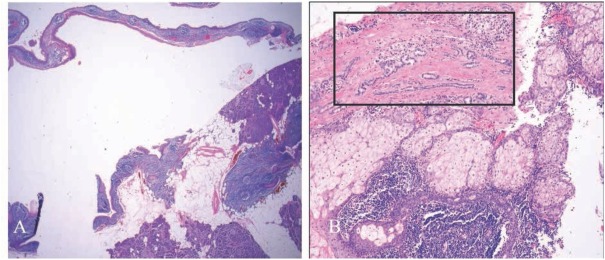
Histopathological examination . The tumor showed a benign cyst lined by mature squamous epithelium and lymphoid structure (H & E stain, X 10). In peripheral portion of the tumor, mature sebaceous glands (black box) are admixed with atrophied pancreatic acini (H & E stain, X 100).

Laparoscopic distal pancreatectomy was performed. A 4 cm x 4 cm-sized well-marginated mass was noted in the pancreas tail. The cystic mass contained cheese-like tenacious material and a 2cm x 2cm-sized soft inner mass. Histologically, the mass contained mature squamous epithelium, lymphoid structure, sebaceous glands and atrophied pancreatic acini (Fig 2). Pathology confirmed it to be a mature cystic teratoma (Fig 2). The patient recovered uneventfully and discharged at postoperative seventh day.

Ethical statements: No ethical approval was needed for this study as it is a retrospective case report.

Patient’s Consent: Written informed consent was obtained from the patient to include images in the article.

## 3 Discussion

Teratomas are germ cell originated neoplasms and are classified as mature or immature type based on the presence of immature neuroectodermal element [8]. Common sites of occurrence are ovary and testes. The pancreas is the rarest site of presentation [9].

Teratomas are classified as “mature” and “immature” teratomas, which replace the terms “benign” and “malignant” teratomas, respectively. The term immature teratoma is used for tumors containing primitive neuroectodermal, endodermal or mesodermal tissues [10]. Each of these histologies may present alone or in combination with others. A mature teratoma consists of an adult-type tumor with well-differentiated elements, while an immature teratoma consists of elements with only partial somatic differentiation, similar to those seen in embryonic or fetal tissue [11].

Differential radiologic diagnosis between benign and malignant teratomas is sometimes difficult, especially when the mature teratoma has ruptured. Malignant teratomas are commonly large mass lesions, and metastasis is evidence of malignancy. In malignant teratomas, CT or MR imaging may reveal lesions with spiculated borders, thick capsules, heterogeneous contents, fat plane obliteration around the tumor, or direct invasion into the adjacent structures with or without effusion [12].

Following the description of a case of a mature cystic teratoma of the pancreas by Kerr in 1918 [9, 13], the characteristics of these tumors have been identified in several case studies. Pancreatic mature cystic teratomas usually develop at a young age, with a mean age at diagnosis of 34.7 years (range 2–74 years). There is also a slight male preponderance in reported cases (59% men, 41% women).

Considering pure benign nature of mature cystic teratomas, resection could be avoided if accurate diagnoses are made. Preoperative diagnostic imaging, however, is challenging and malignant potential cannot be ruled out [7]. From a surgical point of view, definitive treatment should be complete surgical resection.

## 4 Conclusion

Mature cystic teratomas of the pancreas are extremely rare disease entities with a pure nature of benign neoplasm. Preoperative workup including imaging and cytology to draw an accurate diagnosis is challenging. Surgical resection is the standard of care.
